# Changes in the temporomandibular joint clicking and pain disorders after orthognathic surgery: Comparison of orthodontics-first approach and surgery-first approach

**DOI:** 10.1371/journal.pone.0238494

**Published:** 2020-09-04

**Authors:** Ying Zhai, Jeong Joon Han, Seunggon Jung, Min-Suk Kook, Hong-Ju Park, Hee-Kyun Oh

**Affiliations:** 1 Graduate Dental School, Chonnam National University, Gwangju, Republic of Korea; 2 Department of Oral and Maxillofacial Surgery, School of Dentistry, Dental Science Research Institute, Chonnam National University, Gwangju, Republic of Korea; 3 Hard-tissue Biointerface Research Center, School of Dentistry, Chonnam National University, Gwangju, Republic of Korea; Thamar University, Faculty of Dentistry, YEMEN

## Abstract

The purposes of this study were to investigate the influence of the orthodontics-first approach (OFA) and surgery-first approach (SFA) on changes in the signs and symptoms of temporomandibular joint disorders (TMDs) and to compare pre- and postoperative orthodontic treatment duration and total treatment duration between the two approaches. This retrospective study recruited 182 adult patients with malocclusions treated with OFA and SFA and recorded variables such as age, gender, skeletal classification, and signs and symptoms of TMD (clicking and pain disorders) before the start of the surgical-orthodontic treatment and after surgery. Changes in the signs and symptoms of TMD and treatment duration were evaluated within each approach and compared between two approaches. A binary logistic regression was performed to assess the influence of the variables on the postoperative signs and symptoms of TMD. There were no significant postoperative changes in temporomandibular joint (TMJ) pain for OFA and SFA, whereas a significant reduction was found in TMJ clicking after surgery for both approaches. According to binary logistic regression, the type of surgical-orthodontic treatment (OFA or SFA) was not a significant risk factor for postoperative TMJ clicking and pain, and the risk of postoperative TMJ clicking and pain was significant only when TMJ clicking (OR = 10.774, *p* < 0.001) and pain (OR = 26.876, *p* = 0.008) existed before the start of the entire treatment, respectively. With regard to the treatment duration, SFA (21.1 ± 10.3 months) exhibited significantly shorter total treatment duration than OFA (34.4 ± 11.9 months) (*p* < 0.001). The results of this study suggest that surgical-orthodontic treatment using SFA can be a feasible option of treatment for dentofacial deformities based on the equivalent effect on TMD and shorter overall treatment period compared to conventional surgical-orthodontic treatment using OFA.

## Introduction

Temporomandibular disorders (TMDs) are a series of clinical problems that affect the temporomandibular joint (TMJ), masticatory musculatures, the surrounding bony and soft tissue structures, or multiple combinations of these. Common signs and symptoms of TMD include TMJ sounds and pain during mandibular function, headache, facial and neck pain, and limitation in the range of mandibular movements. The etiology of TMD is generally known as complicated and multifactorial [1]. Based on the relevant literature, neuromuscular factors, mechanical and structural factors, and psychosocial factors have been suggested as a source of TMD [2–4]. Several reviews indicate various occlusal factors contribute to development of TMD signs and symptoms, and reported that prevalence of TMD symptoms in patients with dentofacial deformities is higher than that in the general population [3–5].

Surgical-orthodontic treatment is a common treatment protocol for patients with dentofacial deformities, and orthognathic surgery is performed to reposition the maxilla and/or mandible to a more balanced position. Through orthognathic surgery, dental and skeletal harmony can be achieved, and patients can obtain improvement in masticatory function and esthetics [3, 6, 7]. In the TMJ, the positional changes of the mandibular condyle, which is one of the main anatomical structures of the TMJ, usually occur and the relationship between the condyle and surrounding musculature may change after orthognathic surgery [8]. However, there is still not good agreement about whether surgical-orthodontic treatment will improve or aggravate the signs and symptoms of TMD, or have no significant effect on them. Several previous studies have found a beneficial association between orthognathic surgery and TMD with the reduction of the prevalence of postoperative TMD symptomatic patients [9–13]. On the contrary, some studies reported that the positional changes of the condyle in the glenoid fossa during orthognathic surgery may cause TMD symptoms, even causing further deleterious effects on the TMJ and thus worsening pre-existing symptoms and dysfunction [14, 15].

In recent decades, most surgical-orthodontic treatments were performed using the orthodontics-first approach (OFA), which consists of preoperative orthodontics, orthognathic surgery, and postoperative orthodontics. Recently, however, the application of the surgery-first approach (SFA), with no or minimal preoperative orthodontics, has increased due to several advantages. In SFA, the jaw bone is repositioned to a more balanced and ideal position relying on the advanced three-dimensional plan through the surgery at the initial stage of treatment. Current literature have reported on the shorter treatment duration [7, 16–18], earlier esthetic improvement [19], more favorable quality of life [20, 21], similar stability [22–24], and equivalent changes of condylar position in SFA [25]. However, considering the complexity of the diagnosis and treatment planning, and lack of initial occlusal adjustment during the preoperative orthodontic treatment in SFA, a possible limitation of this approach may be associated with a higher risk of postoperative complications, such as new onset or deterioration of TMD signs and symptoms. In contrast to OFA, which has been extensively investigated for the effect on TMD [9, 10, 14, 26–28], few previous reports have examined the signs and symptoms of TMD in SFA.

Therefore, the aims of the present study were to investigate the influence of OFA and SFA on signs and symptoms of TMD, and to compare pre- and postoperative orthodontic treatment duration and total treatment duration between the two approaches. It was hypothesized that: (1) there is a difference between the prevalence of TMD signs and symptoms in OFA and SFA groups; (2) SFA can shorten total treatment duration significantly, compared to OFA.

## Materials and methods

The authors designed and implemented a retrospective cohort study. The study population was composed of all consecutive patients who received surgical-orthodontic treatment from January 2009 to December 2016 at the Department of Oral and Maxillofacial Surgery in Chonnam National University Dental Hospital (CNUDH). All of the patients underwent orthognathic surgery with OFA or SFA. Exclusion criteria were patients with syndromes or craniofacial deformities, such as cleft lip and palate, previous history of orthognathic surgery, the place of orthodontic treatment performed other than the same institution (Department of Orthodontics, CNUDH), and no complete orthodontic treatment durations record. The patients with minimum preoperative orthodontic treatment, which could be included in the SFA in a broad sense, were also excluded. The SFA group included only patients who received no preoperative orthodontic treatment. Due to the retrospective nature of the study and de-identifying the records of patients before the start of the study, it was exempted from approval of the institutional review board of the Chonnam National University Dental Hospital (CNUDH-EXP-2020-019).

To determine which approach to proceed with surgical-orthodontic treatment, the patient’s preferences were first reflected after explaining both OFA and SFA to the patient. When the patient preferred SFA, postoperative orthodontic treatment including dental alignment, incisor decompensation, and arch coordination was predicted and simulated using laboratory procedure and model surgery, and the construction of surgical occlusion was performed. After this diagnosis process, the following criteria were considered to determine whether SFA could be applied or not: 1) good predictability, 2) three or more occlusal stop between the upper and lower arches, 3) mild to moderate curve of Spee or vertical problem, 4) no or mild transverse discrepancy [29].

Regarding surgical procedures, the correction of the maxilla was performed using conventional Le Fort I osteotomy or a two- or three-piece maxillary osteotomy for all patients requiring bimaxillary surgery. For the mandibular surgery, the bilateral sagittal split ramus osteotomy or unilateral sagittal split ramus osteotomy was performed. After splitting of the mandible into the proximal and distal segments, bony interferences between the mandibular segments were removed. The condyle was manually guided to the superior and anterior position within the glenoid fossa, which was regarded as the same condylar position when the centric relation was captured in the preoperative preparation. Then, fixation of the mandibular segments was performed. Postoperative maxillomandibular fixation was accomplished with a surgical splint for 2 or 3 weeks, and mouth-opening exercises were started after 3 weeks of surgery to gain adequate mouth-opening ability greater than 40 mm. All subjects received postoperative orthodontic treatment with or without preoperative orthodontic treatment in one center (Department of Orthodontics, CNUDH), following the complete treatment plan.

The data of subjects were summarized according to the age at time of surgery, gender, skeletal classifications, and signs and symptoms of TMD. Skeletal classifications dividing the subjects into three groups (class I, II and III) were analyzed using lateral cephalograms. The clinical examination of TMJs consisted of the evaluation of TMJ clicking and pain disorders, including arthralgia and muscular disorders (myalgia), and it was performed before starting surgical-orthodontic treatment, after preoperative orthodontic treatment (for the OFA group), and more than 6 months after surgery. TMJ clicking, that was reported by a patient, was confirmed by an examiner from the palpation of the TMJ during the opening and closing of the mouth. Arthralgia was confirmed when the patients reported pain in TMJ in combination of familiar pain with TMJ palpation or range of motion. Myalgia diagnosis was confirmed when the patients reported pain in the masticatory muscles in combination with familiar pain in the masticatory muscles with either muscle palpation or maximum opening. With regard to the signs and symptoms of TMD, the patients were divided into four groups according to assessment of changes in signs and symptoms: (1) pre o → post x: patients with pre-treatment symptoms and whose symptoms disappeared after surgery; (2) pre o → post o; patients with positive symptoms before the start of the treatment and after surgery; (3) pre x → post o: asymptomatic patients developing symptoms after surgery; and (4) pre x → post x: asymptomatic patients before the start of the treatment and after surgery. The treatment duration was recorded as the preoperative orthodontic treatment duration and the postoperative orthodontic treatment duration.

The data were analyzed using SPSS statistics 25.0 for Windows (SPSS IBM, New York, USA). To compare the differences in demographic and clinical characteristics between OFA and SFA groups, the Chi-square test or independent *t* test was performed. The agreement between pre-treatment and postoperative TMD symptoms in each approach was determined with the McNemar test. The difference in prevalence of TMD signs and symptoms between OFA and SFA groups was analyzed using the Chi-square test. Changes in the TMD signs and symptoms depending on the variables of patients were presented using the descriptive statistics. To determine the influencing factors for postoperative TMD signs and symptoms, a binary logistic regression analysis was performed for variables including age, gender, skeletal classification, pre-treatment TMD signs and symptoms, and type of surgical-orthodontic treatment approach (SFA or OFA). For the treatment durations, pre- and post-operative orthodontic treatment duration and total treatment durations were presented by the mean with standard deviation, median, minimum, and maximum values, and compared between OFA and SFA groups using an independent *t* test. The correlations of treatment durations were evaluated using Pearson’s correlation analysis. To evaluate treatment duration among three skeletal classifications, one-way analysis of variance was performed for each group. The significance level was set at *p* < 0.05.

## Results

### Demographic and clinical characteristics of all the patients in OFA and SFA groups

A total of 182 patients (100 males and 82 females; mean age, 22.5 ± 3.8 years; age range, 17–40 years) who met inclusion and exclusion criteria were recruited for the study. Demographics and clinical characteristics of the study sample are summarized in Table 1. Of the 182 patients who received surgical orthodontic treatment, 116 patients with OFA consisted of 62 males (53.4%) and 54 females (46.6%), and 66 with SFA included 38 males (57.6%) and 28 females (42.4%) (*p* = 0.591). There was significant difference in mean age between the two groups (OFA group, 23.3 ± 3.8 years; SFA group, 21.3 ± 3.3 years) (*p* = 0.001). The proportion between young patients (age <21 years) and older patients (age ≥21 years) also exhibited a statistically significant difference (*p* = 0.015). 87 patients (75.0%) of 125 elderly patients received surgical-orthodontic treatment using OFA, whereas SFA was more used for young patients (n = 28, 42.4%). Regarding the variables of skeletal classifications, there were no significant differences in the percentages of patients between OFA and SFA groups (*p* = 0.087). Of the 116 patients in the OFA group and 66 patients in the SFA group, the patients with skeletal class III accounted for 75.0% (n = 87) in the OFA group and 87.9% (n = 58) in the SFA group.

**Table 1:** Demographic and clinical characteristics of study sample.

	OFA (n = 116)	SFA (n = 66)	*p* value
Gender, n (%)			0.591*
male	62 (53.4)	38 (57.6)	
female	54 (46.6)	28 (42.4)	
Age (year), mean ± SD	23.3 ± 3.8	21.3 ± 3.3	0.001†
age distribution, n (%)			0.015*
<21 years	29 (25.0)	28 (42.4)	
≥21years	87 (75.0)	38 (57.6)	
Skeletal classification, n (%)			0.087*
class Ⅰ	13 (11.2)	5 (7.6)	
class Ⅱ	16 (13.8)	3 (4.5)	
class Ⅲ	87 (75.0)	58 (87.9)	

Abbreviation: OFA, orthodontics-first approach; SFA, surgery-first approach; SD, standard deviation.

* By Chi-square test

† By independent *t* test.

### TMD evaluation

Compared to the pre-treatment TMJ clicking, the both OFA and SFA groups exhibited a significant reduction in TMJ clicking after surgery (OFA, *p* < 0.001; SFA, *p* = 0.004) (Table 2). Of the 38 patients with pre-treatment clicking in OFA group, 28 patients (73.7%) exhibited improvement in the clicking after surgery. On the other hand, 4 of 78 patients (5.1%) without pre-treatment TMJ clicking in the OFA group developed clicking postoperatively. In the SFA group, the clicking disappeared postoperatively in 9 of 11 patients (81.8%) with pre-treatment TMJ clicking, and none of those who did not have it preoperatively had developed it by the follow-up over 6 months. With regard to the TMJ pain, there was no statistically significant changes for both OFA and SFA groups (OFA, *p* = 0.999; SFA, *p* = 0.125) (Table 3). Before the surgical-orthodontic treatment, 5 patients in OFA group (arthralgia, 2 patients; myalgia, 2 patients; both arthralgia and myalgia, 1 patient) and 5 patients in SFA group (arthralgia, 4 patients; both arthralgia and myalgia, 1 patients) exhibited pain disorders. Among the patients with pre-treatment TMJ pain disorders, 4 patients (80.0%; arthralgia, 1 patient; myalgia, 2 patients; both arthralgia and myalgia, 1 patient) in the OFA group and 4 patients (80.0%; arthralgia, 3 patients; both arthralgia and myalgia, 1 patient) in the SFA group had an improvement of TMJ pain 6 months after surgery. 3 of 111 patients (2.7%) without pre-treatment TMJ pain in OFA group exhibited postoperative TMJ pain (arthralgia, 2 patients; myalgia, 1 patient), while there were no patients in the SFA group who exhibited newly developed TMJ pain after surgery. In the comparison of pre-treatment and postoperative prevalence of TMJ symptoms between OFA and SFA groups, the SFA group showed significantly lower pre-treatment (*p* = 0.019) and postoperative prevalence of TMJ clicking (*p* = 0.038) than the OFA group (Table 4). With respect to the prevalence of TMJ pain, there were no significant differences between two groups (pre-treatment, *p* = 0.353; postoperative, *p* = 0.443). The changes in prevalence of TMJ symptoms depending on various variables (gender, age, and skeletal classification) are shown in Tables 5 and 6. According to binary logistic regression, the risk of postoperative TMJ clicking and pain was significant only when TMJ clicking (OR = 10.774, *p* < 0.001) and pain (OR = 26.876, *p* = 0.008) existed before treatment, respectively (Table 7).

**Table 2:** Changes of TMJ clicking according to the approaches in surgical-orthodontic treatment.

Subject	After preoperative orthodontic treatment			Postoperatively		
	No clicking	Clicking	*p* value*	No clicking	Clicking	*p* value*
OFA			0.143			< 0.001
Without pre-treatment clicking (n = 78)	73 (93.6%)	5 (6.4%)		74 (94.9%)	4 (5.1%)	
With pre-treatment clicking (n = 38)	12 (31.6%)	26 (68.4%)		28 (73.7%)	10 (26.3%)	
SFA						0.004
Without pre-treatment clicking (n = 55)				55 (100.0%)	0 (0.0%)	
With pre-treatment clicking (n = 11)				9 (81.8%)	2 (18.2%)	

Abbreviation: TMJ, temporomandibular joint; OFA, orthodontics-first approach; SFA, surgery-first approach.

* By McNemar test.

**Table 3:** Changes of TMJ pain disorders according to the approaches in surgical-orthodontic treatment.

Subject	After preoperative orthodontic treatment			Postoperatively		
	No pain	Pain	*p* value*	No pain	Pain	*p* value*
OFA			0.999			0.999
Without pre-treatment pain (n = 111)	110 (99.1%)	1 (0.9%)		108 (97.3%)	3 (2.7%)	
With pre-treatment pain (n = 5)	2 (40.0%)	3 (60.0%)		4 (80.0%)	1 (20.0%)	
SFA						0.125
Without pre-treatment pain (n = 61)				61 (100.0%)	0 (0.0%)	
With pre-treatment pain (n = 5)				4 (80.0%)	1 (20.0%)	

Abbreviation: TMJ, temporomandibular joint; OFA, orthodontics-first approach; SFA, surgery-first approach.

* By McNemar test.

**Table 4:** Comparison of pre-treatment and postoperative prevalence of TMJ symptoms between orthodontics-first approach and surgery-first approach groups.

	Comparison between two groups (*p* value*)	
	Pre-treatment	6-month postoperatively
TMJ clicking	0.019	0.038
Pain disorders	0.353	0.443

Abbreviation: TMJ, temporomandibular joint.

* By Chi-square test.

**Table 5:** Changes in TMJ clicking before the start of the surgical-orthodontic treatment and after surgery.

		OFA (n = 116)				SFA (n = 66)			
		pre o → post x	pre o → post o	pre x → post o	pre x → post x	pre o → post x	pre o → post o	pre x → post o	pre x → post x
Gender									
male	n	14	7	0	41	7	0	0	31
	%	12.1%	6.0%	0.0%	35.3%	10.6%	0.0%	0.0%	47.0%
female	n	14	3	4	33	2	2	0	24
	%	12.1%	2.6%	3.4%	28.4%	3.0%	3.0%	0.0%	36.4%
Age									
<21years	n	7	2	2	18	2	1	0	25
	%	6.0%	1.7%	1.7%	15.5%	3.0%	1.5%	0.0%	37.9%
≥21years	n	21	8	2	56	7	1	0	30
	%	18.1%	6.9%	1.7%	48.3%	10.6%	1.5%	0.0%	45.5%
Skeletal classification									
class I	n	4	0	2	7	2	0	0	3
	%	3.4%	0.0%	1.7%	6.0%	3.0%	0.0%	0.0%	4.5%
class II	n	4	2	1	9	0	0	0	3
	%	3.4%	1.7%	0.9%	7.8%	0.0%	0.0%	0.0%	4.5%
class III	n	20	8	1	58	7	2	0	49
	%	17.2%	6.9%	0.9%	50.0%	10.6%	3.0%	0.0%	74.2%

Abbreviation: TMJ, temporomandibular joint; OFA, orthodontics-first approach; SFA, surgery-first approach; pre o → post x, patients with pre-treatment symptoms and whose symptoms disappeared after surgery; pre o → post o, patients with the positive symptoms before the start of the treatment and after surgery; pre x → post o, asymptomatic patients developing symptoms after surgery; pre x → post x: asymptomatic patients before the start of the treatment and after surgery.

**Table 6:** Changes in TMJ pain disorders before the start of the surgical-orthodontic treatment and after surgery.

		OFA group (n = 116)				SFA group (n = 66)			
		pre o → post x	pre o → post o	pre x → post o	pre x → post x	pre o → post x	pre o → post o	pre x → post o	pre x → post x
Gender									
male	n	1	1	2	58	2	1	0	35
	%	0.9%	0.9%	1.7%	50.0%	3.0%	1.5%	0.0%	53.0%
female	n	3	0	0	51	2	0	0	26
	%	2.6%	0.0%	0.0%	44.0%	3.0%	0.0%	0.0%	39.4%
Age									
<21years	n	2	1	0	26	1	1	0	26
	%	1.7%	0.9%	0.0%	22.4%	1.5%	1.5%	0.0%	39.4%
≥21years	n	2	0	2	83	3	0	0	35
	%	1.7%	0.0%	1.7%	71.6%	4.5%	0.0%	0.0%	53.0%
Skeletal classification									
class I	n	1	0	1	11	1	0	0	4
	%	0.9%	0.0%	0.9%	9.5%	1.5%	0.0%	0.0%	6.1%
class II	n	0	0	1	15	0	0	0	3
	%	0.0%	0.0%	0.9%	12.9%	0.0%	0.0%	0.0%	4.5%
class III	n	3	1	1	82	3	1	0	54
	%	2.6%	0.9%	0.9%	70.7%	4.5	1.5%	0.0%	81.8%

Abbreviation: TMJ, temporomandibular joint; OFA, orthodontics-first approach; SFA, surgery-first approach; pre o → post x, patients with pre-treatment symptoms and whose symptoms disappeared after surgery; pre o → post o, patients with the positive symptoms before the start of the treatment and after surgery; pre x → post o, asymptomatic patients developing symptoms after surgery; pre x → post x: asymptomatic patients before the start of the treatment and after surgery.

**Table 7:** Results of the logistic regression analysis to determine significant variables affecting postoperative TMJ clicking and pain.

	Postoperative TMJ clicking		Postoperative TMJ pain	
	OR (95% CI of OR)	*p* value	OR (95% CI of OR)	*p* value
Age	0.959 (0.830–1.107)	0.566	0.865 (0.593–1.261)	0.451
Gender	0.508 (0.162–1.590)	0.245	7.108 (0.543–93.089)	0.135
Skeletal classification				
class I (reference)	1	0.875	1	0.441
class II	1.258 (0.149–10.637)	0.833	1.344 (0.061–29.526)	0.851
class III	0.837 (0.144–4.867)	0.843	0.287 (0.022–3.838)	0.347
Pre-treatment clicking	10.774 (3.064–37.882)	< 0.001	1.434 (0.181–11.346)	0.733
Pre-treatment pain	0	0.999	26.876 (2.397–301.324)	0.008
Treatment approach (OFA or SFA)	2.955 (0.568–15.371)	0.198	3.393 (0.289–39.786)	0.331

TMJ, temporomandibular joint; OR, odds ratio; CI: confidence interval; OFA, orthodontics-first approach; SFA, surgery-first approach.

For OFA group, we evaluated the changes of TMJ clicking and pain disorders during the preoperative orthodontic treatment. Among the 38 patients with pre-treatment clicking, TMJ clicking disappeared in 12 patients (31.6%), although 26 patients (68.4%) still exhibited TMJ clicking after preoperative orthodontic treatment. In addition, 5 patients who had no TMJ clicking before the start of the treatment also showed a newly developed TMJ clicking after preoperative orthodontic treatment. However, there were no statistically significant changes in TMJ clicking and pain disorders during the preoperative orthodontic treatment period (TMJ clicking, *p* = 0.143; pain disorders, *p* = 0.999).

### Total treatment duration

The analysis of treatment durations in OFA and SFA groups are shown in Table 8. The mean preoperative orthodontic duration in the OFA group was 22.4 ± 10.6 months. The total treatment duration of OFA and SFA groups was 34.4 ± 11.9 months and 21.1 ± 10.3 months, respectively. Though postoperative orthodontic treatment duration was significantly longer in the SFA group (21.1 ± 10.3 months) than in the OFA group (12.0 ± 7.4 months) (*p* < 0.001), the SFA group exhibited significantly shorter total treatment duration than the OFA group (*p* < 0.001).

**Table 8:** Comparison of treatment durations between orthodontics-first approach and surgery-first approach groups.

	OFA group				SFA group				
	Mean ± SD	Med	Min	Max	Mean ± SD	Med	Min	Max	*p* value*
Preop-OTD	22.4 ± 10.6	20.0	6.0	71.0	0.0	0.0	0.0	0.0	< 0.001
Postop-OTD	12.0 ± 7.4	10.0	4.0	45.0	21.1 ± 10.3	18.0	8.0	51.0	< 0.001
Total-TD	34.4 ± 11.9	32.0	17.0	75.0	21.1 ± 10.3	18.0	8.0	51.0	< 0.001

Data are presented in months.

Abbreviation: OFA, orthodontics-first approach; SFA, surgery-first approach; SD, standard deviation; Med, median value; Min, minimum value; Max, maximum value; Preop-OTD, preoperative orthodontic treatment duration; Postop-OTD, postoperative orthodontic treatment duration; Total-TD, total treatment duration.

* By independent *t* test.

In the correlation analysis between pre- or postoperative orthodontic treatment duration and total treatment duration, the positive correlation between preoperative and total treatment durations in OFA group was stronger (r = 0.712, *p* < 0.001), comparing the correlation between postoperative and total treatment durations (r = 0.438, *p* < 0.001). Table 9 represents the treatment duration with OFA and SFA to evaluate the skeletal classifications as an independent variable. Skeletal classifications had no significant influence on the preoperative, postoperative, and total treatment duration in OFA group. However, in SFA group, there were significant differences in total treatment duration among three skeletal classifications (*p* = 0.028; class I vs class II, *p* = 0.128; class II vs class III, *p* = 0.024; class III vs class I, *p* = 0.999), where the average duration of total treatment in SFA groups was 21.4 ± 13.7 months for class I, 36.3 ± 5.0 months for class II, and 20.3 ± 9.7 months for class III patients.

**Table 9:** Comparison of treatment durations among skeletal classifications in orthodontics-first approach and surgery-first approach groups.

	OFA group							SFA group						
	class I		class II		class III		*p* value*	class I		class II		class III		*p* value*
	Mean	SD	Mean	SD	Mean	SD		Mean	SD	Mean	SD	Mean	SD	
Preop-OTD	20.9	5.3	22.6	9.6	22.5	11.4	0.873	0.0	0.0	0.0	0.0	0.0	0.0	-
Postop-OTD	9.3	5.2	10.9	5.1	12.6	7.9	0.271	21.4	13.7	36.3	5.0	20.3	9.7	0.028
Total-TD	30.2	7.6	33.6	9.4	35.1	12.8	0.374	21.4	13.7	36.3	5.0	20.3	9.7	0.028

Data are presented in months.

Abbreviation: OFA, orthodontics-first approach; SFA, surgery-first approach; SD, standard deviation; Preop-OTD, preoperative orthodontic treatment duration; Postop-OTD, postoperative orthodontic treatment duration; Total-TD, total treatment duration.

* By one-way analysis of variance.

## Discussion

To our knowledge, this is the first study to evaluate the changes of TMD signs and symptoms in patients with dentofacial deformities who were treated by OFA and SFA. The prevalence of diagnostic TMJ clicking in OFA and SFA groups decreased after surgical-orthodontic treatment. According to results of logistic regression, the type of surgical-orthodontic treatment (OFA or SFA) was not a significant risk factor for postoperative TMJ clicking and pain. Total treatment duration in the SFA group was significantly shorter than that in the OFA group. Thus, the first hypothesis would be rejected, but the second confirmed.

In our study, the prevalence of pre-treatment TMJ clicking in patients with dentofacial deformities was 32.8% in the OFA group and 16.7% in the SFA group, and the prevalence of pre-treatment TMJ pain was 4.3% in the OFA group and 7.6% in the SFA group. In OFA group, the prevalence of preoperative TMJ symptoms after preoperative orthodontic treatment was 26.7% for TMJ clicking and 3.4% for pain disorders. However, the prevalence of TMD can be affected by race, age, and investigation methods, and a systematic review of Al-Riyami, Cunningham [30] showed that the pre-existing signs and symptoms of TMD in patients who had undergone orthognathic surgery ranged from 5% to 86%. Regarding the factors that predispose TMD symptoms, several authors have reported that female patients with a class II facial profile seem to be an increased risk for preoperative TMD symptoms. However, in the present study, there were no statistical differences in the prevalence of preoperative TMJ symptoms between male and female patients for both OFA and SFA groups. With respect to the effect of skeletal classification on preoperative TMD symptoms, it was difficult to draw a general conclusion due to the relatively small number of patients of class II facial profile in this study. In Asian countries, most patients undergoing surgical-orthodontic treatment have a skeletal class III facial profile, and, in our study, 87 of 116 patients of the OFA group and 58 of 66 patients of the SFA group had a class III facial profile, while only 16 patients of the OFA group and three patients of the SFA group had a class II facial profile preoperatively.

Although there have been previous reports of the effect of orthognathic surgery on TMD, the effect of orthognathic surgery on the signs and symptoms of TMD is still controversial [13, 14, 27, 28, 31]. Westermark, Shayeghi [13] reported that the prevalence of TMD symptoms decreased from 43% to 28% after surgery, although the overall positive effect on TMD was less effective in patients with mandibular retrognathism. Abrahamsson, Henrikson [26] also reported a positive effect in respect of TMD pain in patients who received surgical-orthodontic treatment for correction of dentofacial deformity. In a study using clinical and magnetic resonance imaging findings [32], the disc position was improved by accurate repositioning of the condylar-disc complex after surgical-orthodontic treatment in class II patients. In contrast, in the study by Wolford, Reiche-Fischel [14], no TMJ pain relief was observed after surgery in patients with preoperative pain, and, furthermore, six patients experienced postoperative condylar resorption. They concluded that patients with preexisting TMJ dysfunction are more likely to have worsening of the TMJ dysfunction after orthognathic surgery, especially after mandibular advancement. For SFA, there was only one previous report that studied the effect of SFA on the TMJ [12]. Pelo, Saponaro [12] assessed TMD signs and symptoms in 24 patients who were treated with SFA, and reported a significant improvement or resolution of the TMD signs and symptoms postoperatively in the majority of patients with preoperative TMD. In our study, we assessed the changes in signs and symptoms of TMD after surgical-orthodontic treatment and compared them between OFA and SFA. Although, there was no statistically significant improvement in the prevalence of TMJ pain, 4 of 5 patients with pre-treatment pain in the OFA group and 4 of 5 patients with pre-treatment pain in the SFA group exhibited relief of TMJ pain after surgery. With respect to TMJ clicking, the prevalence of TMJ clicking reduced significantly from 32.8% (38 of 116) before the beginning of the treatment to 12.1% (14 of 116) after surgery in the OFA group, and from 16.7% (11 of 66) to 3.0% (2 of 66) in the SFA group. Clicking disappeared in 73.2% (28 of 38) of pre-treatment symptomatic patients in the OFA group and in 81.82% (9 of 11) in the SFA group.

Despite the positive effect of orthognathic surgery on TMD, several investigators have suggested that postoperative TMJ dysfunction may develop after surgery in patients with no preoperative TMD signs and symptoms [10, 28, 33]. In a study by Karabouta and Martis [33], 3.7% of patients with no TMJ dysfunction preoperatively presented postoperative TMJ dysfunction. Togashi, Kobayashi [28] reported that TMJ signs and symptoms developed in 9.7% of preoperative asymptomatic patients. In our study, 3 of 111 patients (2.7%) with no pre-treatment TMJ pain in the OFA group and none of the patients in the SFA group exhibited postoperative development of TMJ pain. Postoperative clicking developed in 4 of 78 (5.1%) of patients with no pre-treatment clicking in the OFA group, and none in the SFA group. Regarding the predictive factors for postoperative TMD signs and symptoms, pre-existing TMJ clicking and pain were detected as significant factors for postoperative symptoms. Our results are consistent with other previous studies [10, 34]. In a study in which postoperative TMD was assessed in patients with class III malocclusion by Scolozzi, Wandeler [34], anamnestic TMJ clicking and bimaxillary surgery were suggested as clinical predictive factors predisposing to the envelopment or worsening of clinical dysfunction in TMDs. Kretschmer, Baciut [10] also reported that preoperative clicking and preoperative crepitus were significant factors for postoperative symptoms.

The present findings indicate that the average total treatment time with OFA is 34.4 months, which is possibly longer than indicated by results of previous studies, which vary from 21.9 months as found by Jacobson [35] to 32.8 months reported by O'Brien, Wright [36]. This could be influenced by a greater number of appointments or slightly longer appointment interval schedule. No clinically significant association between preoperative and postoperative treatment duration was found, so there was no evidence to suggest that the period of preoperative orthodontic treatment is associated with a decreased or increased postoperative orthodontic treatment time. In the comparison between OFA and SFA, total treatment duration in SFA was significantly shorter than that in OFA, which might be due to the reduction or elimination of pre-surgical orthodontic treatment time [37]. Furthermore, the increase in osteoclastic activities and metabolic changes (regional acceleratory phenomenon) after orthognathic surgery might accelerate orthodontic tooth movement [17]. In a biological evaluation after orthognathic surgery using SFA, Zingler, Hakim [38] reported that the concentration of remodeling factors in the crevicular fluid increased at later postoperative time points and suggested that accelerated orthodontic tooth movement might be related to elevated levels of these factors.

This study has several limitations. First, the total number of patients is not sufficient to draw generalized strong conclusions. Thus, future studies including greater number of patients are needed. In addition, more long-term evaluation is necessary because TMD may develop even after the entire surgical-orthodontic treatment, including postoperative orthodontic treatment. Another limitation of this study is the difference in the age of patients between OFA and SFA groups. This is probably because most of the patients who start the surgical-orthodontic treatment after graduating from high school prefer SFA to receive orthognathic surgery before starting social life or entering college. The age difference between two groups could have contributed to the signs and symptoms of TMJ. Although magnetic resonance imaging (MRI) analysis of patients with TMD was not performed in this study, the evaluation of disc position using MRI may provide a better understanding of the effect of orthognathic surgery on TMJ.

## Conclusions

The type of surgical-orthodontic treatment (OFA or SFA) was not a significant risk factor for postoperative TMJ clicking and pain after orthognathic surgery. Total treatment duration in the SFA group was significantly shorter than that in the OFA group. The results of this study suggest that surgical-orthodontic treatment using SFA can be a feasible option for treatment of dentofacial deformities based on the equivalent effect on TMD and shorter overall treatment period compared to conventional surgical-orthodontic treatment using OFA.
